# Adherence to guideline recommendations for coronary angiography in a poor South-East Asian setting: Impact on short- and medium-term clinical outcomes

**DOI:** 10.1038/s41598-019-55299-0

**Published:** 2019-12-16

**Authors:** Andriany Qanitha, Cuno S. P. M. Uiterwaal, Jose P. S. Henriques, Idar Mappangara, Muzakkir Amir, Sumarsono G. Saing, Bastianus A. J. M. de Mol

**Affiliations:** 1 Department of Cardio-thoracic Surgery, AMC Heart Center, Amsterdam University Medical Center, University of Amsterdam, Amsterdam, The Netherlands; 2 Department of Physiology, Faculty of Medicine, Hasanuddin University, Makassar, Indonesia; 3 Julius Global Health, Julius Center for Health Sciences and Primary Care, University Medical Center Utrecht, Utrecht University, Utrecht, The Netherlands; 4 Department of Cardiology, AMC Heart Center, Amsterdam University Medical Center, University of Amsterdam, Amsterdam, The Netherlands; 5 Department of Cardiology and Vascular Medicine, Faculty of Medicine, Hasanuddin University, Makassar, Indonesia; 6 Makassar Cardiac Center, Catheterization Laboratory Unit, DR. Wahidin Sudirohusodo Hospital, Makassar, Indonesia

**Keywords:** Cardiology, Epidemiology, Prognosis

## Abstract

In South-East Asian populations and particularly in Indonesia, access to coronary angiography (CAG) is limited. We aimed to assess the adherence for undergoing CAG for indicated patients, according to the guideline recommendations. We then examined whether this adherence would have an impact on patients’ short- and medium-term mortality and morbidity. We consecutively enrolled 474 patients with acute and stable coronary artery disease who had indication for CAG at Makassar Cardiac Center, Indonesia from February 2013 to December 2014. We found that adherence to CAG recommendation in poor South-East Asian setting is low. Of 474 recommended patients, only 273 (57.6%) underwent the procedure. Factors for not undergoing CAG were: older age, female gender, low educational and socio-economic status, and insurance type. While reasons for patients refusing CAG and subsequent intervention included fear, symptoms reduction, and lack of trust concerning the procedure benefit. During follow-up (median 19 (IQR 6–39.3) months), 155 (32.7%) patients died, and 259 (54.6%) experienced at least one adverse event. Adherence to CAG recommendation was associated with a significantly lower short- and medium-term mortality, independent of revascularization and other potential confounders. In sub-group analysis, adhered patients “with revascularization” had significantly better outcomes compared to the “non-revascularization” and “not adhere” groups.

## Introduction

Coronary angiography (CAG) is the diagnostic test used to detect and quantify the presence and extent of atherosclerotic coronary artery disease (CAD)^1^. CAG should be available to patients with acute coronary syndrome (ACS) and stable CAD for several purposes: (1) recognition and treatment of acute myocardial infarction – urgent invasive strategy (<2 hours of admission); (2) early invasive strategy (<24 hours), or delayed invasive strategy (<25–72 hours); and (3) risk stratification (elective diagnostic CAG)^2–4^.

Patient with angiographically proven coronary artery disease may undergo revascularization, either by percutaneous coronary intervention (PCI) or coronary artery bypass grafting (CABG)^5^. The SYNTAX score is an angiographic tool developed to grade the complexity and severity of coronary artery lesions^5,6^. This score assists clinicians to select the optimal revascularization strategy^5,6^. A higher SYNTAX score indicates a more complex condition and a worse prognosis in patients undergoing revascularization, especially PCI^5^.

This cohort is the first study to measure the adherence to the guidelines recommendation to perform CAG in a setting with limited resources. The study also intends to demonstrate the short- and medium-term prognostic consequences of the adherence to these guidelines.

## Methods

### Study population

This study was based on a prospective cohort of 474 patients with CAD who were clinically indicated for undergoing CAG, at the Makassar Cardiac Center, Indonesia, between February 2013 and December 2014. This cardiac center is the biggest in Eastern Indonesia, serving the population of South Sulawesi as well as the populations from other regions in Sulawesi and beyond. The study protocol was approved by the Ethics Committee and Institutional Review Board of the Faculty of Medicine, Hasanuddin University Makassar. All methods performed in this study were in accordance with the relevant guidelines and regulations.

All patients were recruited from either the cardiovascular care unit (including intensive care and catheterization lab) or the regular ward. Patients were included if they had been diagnosed with CAD, defined as acute coronary syndrome (ACS) or stable coronary artery disease (SCAD). ACS was defined as unstable angina (UA), non-ST-segment elevation myocardial infarction (NSTEMI), or ST-segment elevation myocardial infarction (STEMI)^7,8^. SCAD was defined as stable angina, including prior ACS, prior coronary revascularization or known significant CAD on angiography without revascularization^9^. All eligible patients gave written informed consent before being enrolled. We excluded all patients who immediately died in the emergency department or intensive cardiovascular care unit before giving informed consent, and all patients in whom coronary angiography had been confirmed as normal (defined as 0% lumen stenosis in all coronary vessels)^10^. Detailed description and flowchart of the study population has been previously reported^11^.

### Data collection

Socio-demographic data, lifestyle, family history of cardiovascular disease (CVD) and clinical profiles were all collected from medical records and questionnaire interviews. We also recorded clinical data from electrocardiography and echocardiography examinations. In patients with symptomatic angina, cardiac enzymes were immediately tested to confirm the clinical diagnosis. Laboratory tests including plasma glucose, lipid profiles, uric acid, and renal and liver function markers were measured within 24 hours of hospital admission. An overnight 8-hour fasting blood sample was taken via venipuncture. All blood samples were analyzed in the hospital laboratory using standardized methods.

### In-hospital and after-discharge management

Management strategies before, during, and after hospitalization including medications, fibrinolysis/thrombolysis, primary and elective PCI, and elective CABG were all recorded. At thirty days from admission, we followed up the adherence to after-discharge and secondary prevention medications in all survivors (n = 406).

### Coronary angiography

As our main determinant, we collected all data on coronary angiography including date and time of hospital admission, indications, contraindications, procedures, findings, and SYNTAX scores.

The recommendation to perform CAG in clinically indicated patients was based on the American College of Cardiology (ACC)/American Heart Association (AHA) guidelines for coronary angiography (1999)^2,3^. Detailed descriptions of the classes of recommendation (COR) and level of evidence (LOE) for CAG procedure are given as supplement (Table S1)^2–4,12^. In the present study, we measured the adherence to the guideline recommendation to perform a coronary angiography as the initial diagnostic tool or as a precondition of an urgent or early invasive treatment, and for the risk stratification purposes, both from patients’ and clinicians’ sides.

We marked the clinical recommendation as classes IA and IB (Table S2 in supplement). Patients who did not consent to undergo CAG were asked about the motivation for their decision.

A trained and experienced catheterization laboratory analyst (SS) and a cardiologist (IM) read all video recordings of the coronary angiograms and analyzed the SYNTAX scores. Significant or obstructive vessel disease was defined as a lesion in a coronary vessel (diameter ≥1.5 mm) with ≥50% reduction in luminal diameter on angiographic assessment^5^. In this study, the SYNTAX scores of the non-significant lesions were also quantified.

### Definitions

The Canadian Cardiology Society (CCS) grading of angina pectoris was used to classify patients with stable angina. We also used the New York Heart Association (NYHA) Functional Classification system to grade heart failure according to the severity of symptoms.

In patients with non-ST-elevation acute coronary syndrome (NSTE-ACS), diagnostic angiography within 24 hours of admission with intent to perform revascularization based on the coronary anatomy findings was defined as the appropriate early invasive strategy^3^. Increased risk for clinical events was defined if one or more of the following features were present: prolonged ongoing pain at rest (>20 minutes), angina at rest with dynamic ST changes ≥1 mm, pulmonary edema, angina with new or worsening mitral regurgitation (MR) murmur, angina with S_3_ or new/worsening rales, or angina with hypotension^2^. Patients with prior revascularization, congestive heart failure (CHF), LVEF < 50%, ventricular arrhythmia, or persistent/recurrent ischemic angina who had an indication for CAG, were considered at high-risk for peri-operative complications^2^.

The Killip classification was used to stratify the risk of mortality in acute myocardial infarction (STEMI and NSTEMI) patients.

### Follow-up

The endpoints of this study were all-cause mortality and the composite outcome of major adverse cardiovascular events (MACE). We actively collected outcomes data during hospitalization, at 30 days, 6 months, and 12 months, and yearly until the end of study period in January 2018. At home visits or phone call interviews, nurses recorded the dates on which patients had experienced adverse events. These included: cardiovascular and non-cardiovascular mortality, CVD (MI, heart failure, and/or stroke) and non-CVD re-hospitalization, and repeat PCI, However, the cause of mortality was largely based on reports from family members. We considered such a report to be cardiovascular death if patients died suddenly at home, or on the way to hospital, or during hospitalization for a cardiovascular cause or having shown a CVD symptom resulting in death.

### Statistical analysis

Means ± standard deviations (SD) were calculated for continuous variables. Data with skewed distribution were presented as median (Q1-Q3). For categorical variables, we calculated numbers (proportions). Differences in continuous variables were estimated using independent t-test, while differences in proportions were compared using Pearson’s or exact Chi-square test. For skewed continuous data, differences were estimated using Mann-Whitney U test.

Patients were divided according to their adherence to CAG recommendation: “adhere” and “not adhere”. We then categorized adhered patients into “revascularization” and “no revascularization” sub-groups; and subsequently, patients with CAG were categorized based on their SYNTAX score (SS): low (≤18), intermediate (19–27), and high (≥28).

Kaplan-Meier curves were constructed to present the cumulative incidence of short- and medium-term all-cause mortality and MACE, with a cut off at 30 days after admission. The log-rank test was used to estimate the difference between: (a) two groups: “Adhere” vs. “Not adhere”; and (b) three groups: “Adhere **with** revasculariztion” vs. “Adhere without revascularization” vs. “Not adhere”. Follow-up time was defined as the time from hospital admission to mortality or censoring, until the end of study period. The survival times of participants, who had moved, given an incorrect address or who, for whatever reason, were unreachable via telephone or home visit were censored at the last date that they were known to be still alive. Survival times of participants who were still alive at the end of follow-up were censored on January 5, 2018.

We applied uni- and multivariable Cox regression analyses to estimate the hazard ratios (HRs) for all-cause mortality and composite MACE between the adhere, revascularization (yes and no), and SYNTAX tertiles (low, intermediate, and high scores) groups vs. “not adhere” group. All hazard ratios were adjusted for: age, gender, socio-economic status, education, hypertension, hyperglycemia on admission, eGFR < 60 mL/min, LVEF < 35%, undergoing revascularization (PCI/CABG), adherence on after-discharge medications, and living on rural area (i.e. distance ≥20 kms from hospital). In our previous study, all these variables are shown to be the independent predictors for mid-term all-cause mortality in patients with coronary artery disease in Indonesia^11^. We considered a 95% confidence interval not including one, with p-value < 0.05 as statistically significant. All statistical analyses were performed with SPSS ver. 23.0 and R studio ver. 1.2.5.

## Results

A total of 474 patients who clinically recommended for undergoing CAG were included in our analysis: 361 (76.2%) patients admitted for ACS, and 113 (23.8%) for stable CAD. Out of 113 patients with stable CAD, 19 (16.8%) were hospitalized for stable angina, 34 (30.1%) for CHF, and 60 (53.1%) were recruited from the catherization lab. The majority of patients (72.8%) were male with a mean age of 58.1 ± 10.7 years. Eventually, 273 (57.6%) patients underwent the procedure: 93 (34.1%) were performed as an early invasive strategy (<24 hours of admission), and the remaining 180 (65.9%) as an elective CAG.

### General characteristics

Table 1 presents the baseline and clinical profiles of the cohort according to the adherence to CAG recommendation. When compared with the “adhere”, patients in “not adhere” group were older, more were female, with a lower educational level and socio-economic status, less often current or former smokers, and fewer had parents with CVD. With respect to clinical presentation, the not-adhered patients had less history of prior MI, CAG, or PCI; more often had diabetes, reduced renal function, and heart failure with NYHA class III and IV; stayed longer in hospital, and more were diagnosed with NSTEMI compared to the adhere group.

**Table 1:** Baseline characteristics and clinical profiles of the study population according to the adherence to CAG recommendation.

Variables	Adhere	Not adhere	Total	p-value
	(n = 273)	(n = 201)	(n = 474)	
Age (years)	57.1 ± 10.0	59.5 ± 11.6	58.1 ± 10.7	0.017
Male sex	220 (80.6)	125 (62.2)	345 (72.8)	<0.001
Previous MI	108 (39.6)	56 (27.9)	164 (34.6)	0.008
Previous CAG	64 (23.4)	5 (2.5)	69 (14.6)	<0.001
Previous PCI	26 (9.5)	1 (0.5)	27 (5.7)	<0.001
**Risk factors**				
Hypertension	201 (73.6)	155 (77.1)	356 (75.1)	0. 385
Diabetes mellitus	73 (26.7)	73 (36.3)	146 (30.8)	0.026
Dyslipidemia	219 (80.2)	148 (73.6)	367 (77.4)	0.09
Current/former smoker	185 (67.8)	111 (55.2)	296 (62.4)	0.005
Parents with CVD	80 (29.3)	42 (20.9)	122 (25.7)	0.039
BMI (kg/m^2^)	24.4 ± 3.2	24.1 ± 3.5	24.3 ± 3.3	0.292
Low education*	43 (15.8)	93 (46.3)	136 (28.7)	<0.001
Low socio-economic status^†^	99 (36.3)	147 (73.1)	246 (51.9)	<0.001
>20 km from hospital (rural)	140 (51.3)	105 (52.5)	245 (51.7)	0.837
**Clinical presentation**				
Stable angina	90 (33.0)	23 (11.4)	113 (23.8)	<0.001
Unstable angina	44 (16.1)	24 (11.9)	68 (14.3)	0.2
Non-ST-elevation MI	27 (9.9)	57 (28.4)	84 (17.7)	<0.001
ST-elevation MI	112 (41.0)	97 (48.3)	209 (44.1)	0.117
LVEF (%)^‡^	48.4 ± 14.3	41.8 ± 13.2	45.8 ± 14.2	<0.001
eGFR < 60 mL/min	67 (24.5)	82 (40.8)	149 (31.4)	<0.001
CHF/Killip class ≥ 2	70 (25.6)	113 (56.2)	183 (38.6)	<0.001
**NYHA**				
Class II	9 (3.3)	11 (5.5)	20 (4.2)	0.244
Class III	27 (9.9)	46 (22.9)	73 (15.4)	<0.001
Class IV	2 (0.7)	10 (5.0)	12 (2.5)	0.004
Onset to admission (hours)§	27 (9–48)	24 (10–48)	25.5 (10–48)	0.678
Length of stay (days)§	6 (0–8)	7 (5–10)	6 (4–9)	<0.001

Values are n (%) or means ± SD. Comparison was performed using independent t-test for continuous variables and Pearson Chi-square test for categorical variables.

*Defined as the highest formal education was junior high/elementary school, no schooling, or illiterate.

^†^Defined as monthly income <Rp.1,810,000- (USD 1 = Rp. 13,500,-). The cut point based on the national average of minimum income for proper life in 2015.

^‡^Echo was examined in 261 patients.

^§^Values are medians (Q1-Q3). Comparison was performed using Mann-Whitney U test.

CAG = coronary angiography; MI = myocardial infarction; PCI = percutaneous coronary intervention; CVD = cardiovascular disease; BMI = body mass index; LVEF = left ventricular ejection fraction; eGFR = estimated glomerular filtration rate; CHF = congestive heart failure; NYHA = New York Heart Association.

### Result 1: Adherence to CAG recommendation

We describe in detail on how our cohort (n = 474) were clinically recommended to undergo a diagnostic CAG as supplement (Table S2). A total of 201 (42.4%) patients did not adhere the recommendation to undergo the procedure; n = 23 (11.4%) in SCAD, n = 81 (40.3%) in NSTE-ACS, and n = 97 (48.3%) in STEMI patients. Of those, 152 (75.6%) participants deliberately refused CAG or coronary revascularization for various reasons.

Detailed reasons and the motivation behind the decisions of patients not undergoing the CAG procedure are listed in Table 2. Due to limited resources (i.e. poor transportation, complicated administration, limited cardiologists, ect.), 49 (24.4%) ACS patients who should be treated with an urgent/early invasive strategy did not receive the recommended treatment: 30 NSTE-ACS patients with refractory angina, hemodynamic/ECG instability, or at high risk for adverse events; 16 STEMI patients should have had primary/rescue PCI, or with persistent angina; and three STEMI patients with cardiogenic shock (see Table S2).

**Table 2:** Reasons not to undergo diagnostic coronary angiography (CAG) for indicated patients (n = 201).

Reasons/motivations	Frequency (%)
High costs without possibility to cover these expenses (no funds or insurance)	1 (0.5)
Fear of side effects of the procedure that will be debilitating, cause disability, and life-threatening	28 (13.9)
Fear that the procedure is a major/big surgical procedure and patient is not ready for all the potential risks	22 (10.9)
Family’s refusal for the patient to undergo the procedure	5 (2.5)
Reduced symptoms on current medication and patient is convinced that additional procedures or treatments are not necessary	46 (22.9)
Denial of a heart disease (e.g. belief that symptoms are caused by other reasons)	1 (0.5)
Personal uncertainty concerning the beneficial impact on clinical outcomes or quality of life	23 (11.4)
Preference to the procedure at a larger tertiary center (the National Heart Center in Jakarta)	1 (0.5)
Other reasons (e.g. older age; preference for traditional medications or natural healing; relatives/friends’ negative information, belief, etc.)	15 (7.5)
Unknown or personal reasons	10 (5.0)
Constrained by limited resources (i.e. poor transportation, complicated administration, or limited cardiologists)	49 (24.4)
**Total**	**201** (**100.0**)

### Result 2: Follow-up and outcomes

The median follow-up of the cohort was 19 (IQR 6–39.3) months. During the observation period, 155 (32.7%) patients died, 259 (54.6%) experienced adverse events, and seven (1.5%) participants were lost to follow-up.

Table 3 lists in detail patients’ clinical outcomes at short- and mid-term follow-up – with a cut off at 30 days after admission – across the “adhere” (with and without revascularization) and “not adhere” groups. At 30 days, there was a considerably higher rate of cardiovascular mortality in the not-adhere compared with the revascularization and non-revascularization groups (23.9% vs. 2.7% vs. 6.9%, respectively; p < 0.001). Over 30 days up to the end of follow-up period, we also observed that more not-adhered patients died due to CVD compared with the two counterparts (22.4% vs. 11.5% vs. 20.6%), although borderline significant (p = 0.054). Interestingly, in mid-term follow-up, there were more revascularization (18.6%) patients re-admitted to hospital due to CVD, compared to non-revascularization (12.5%) and not-adhere (8.0%) groups (p = 0.021).

**Table 3:** Follow-up results: Clinical outcomes.

Clinial outcomes	Adhere		Not adhere (n = 201)	Total (n = 474)	p-value
	With Revascularization (n = 113)	No Revascularization (n = 160)			
**≤30 days**					
CVD death	3 (2.7)	11 (6.9)	48 (23.9)	62 (13.1)	<0.001
CVD re-hospitalization*	9 (8.0)	17 (10.6)	4 (2.0)	30 (6.3)	0.003
First PCI/CABG	24 (21.2)	N/A	N/A	24 (5.1)	N/A
Repeated PCI/CABG	5 (4.4)	N/A	N/A	5 (1.1)	N/A
**>30 days up to the end of follow-up**					
CVD death	13 (11.5)	33 (20.6)	45 (22.4)	91 (19.2)	0.054
Non-CVD death	0 (0.0)	1 (0.6)	1 (0.5)	2 (0.4)	0.718
CVD re-hospitalization*	21 (18.6)	20 (12.5)	16 (8.0)	57 (12.0)	0.021
Non-CVD hospitalization	2 (1.8)	0 (0.0)	0 (0.0)	2 (0.4)	0.040
First PCI/CABG^†^	14 (12.4)	5 (3.1)	4 (2.0)	23 (4.9)	<0.001
Repeated PCI/CABG^†^	9 (8.0)	N/A	N/A	9 (1.9)	N/A
**At 30-day follow-up**^**‡**^	(n = 110)	(n = 149)	(n = 153)	(n = 412)	
Adherence to post-discharge medication	76 (69.1)	61 (40.9)	42 (27.5)	179 (43.4)	<0.001

Values are n (%). Differences were estimated using Pearson’s or exact Chi-square test.

*Defined as re-hospitalization due to myocardial infarction, heart failure, stroke, and stent thrombosis.

^†^Measured at ≥ 6 months after discharge.

^‡^Measured only for survivors.

CVD = cardiovascular disease; PCI = percutaneous coronary intervention; CABG = coronary artery bypass grafting.

**Figure 1:**
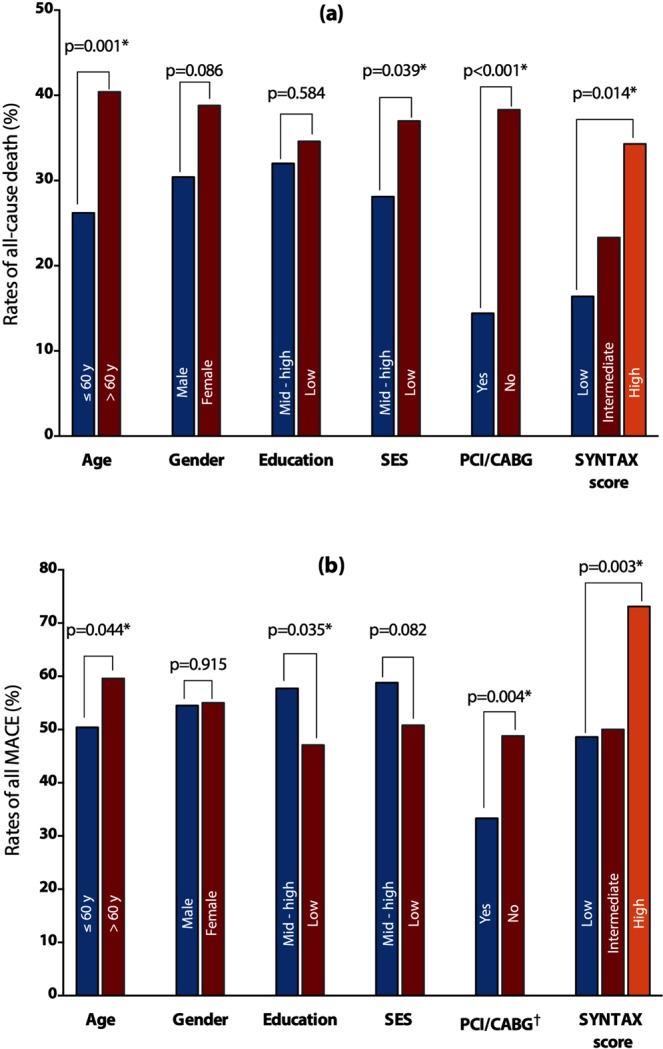
The bar charts show the effect of major determinants of non-adherence, revascularization (PCI/CABG), and SYNTAX score tertiles on patients’ clinical outcomes: all-cause death (**a**) and all MACE (**b**). *p < 0.05. ^†^First and repeated revascularization (PCI/CABG) as adverse outcomes were excluded from the analysis. SES = socio-economic status; PCI = percutaneous coronary intervention; CABG = coronary artery bypass grafting.

We present the bar charts showing the impact of major determinants of non-adherence to CAG recommendation, revascularization (yes/no), and SYNTAX tertile groups on patients’ clinical outcomes in Fig. 1.

**Figure 2:**
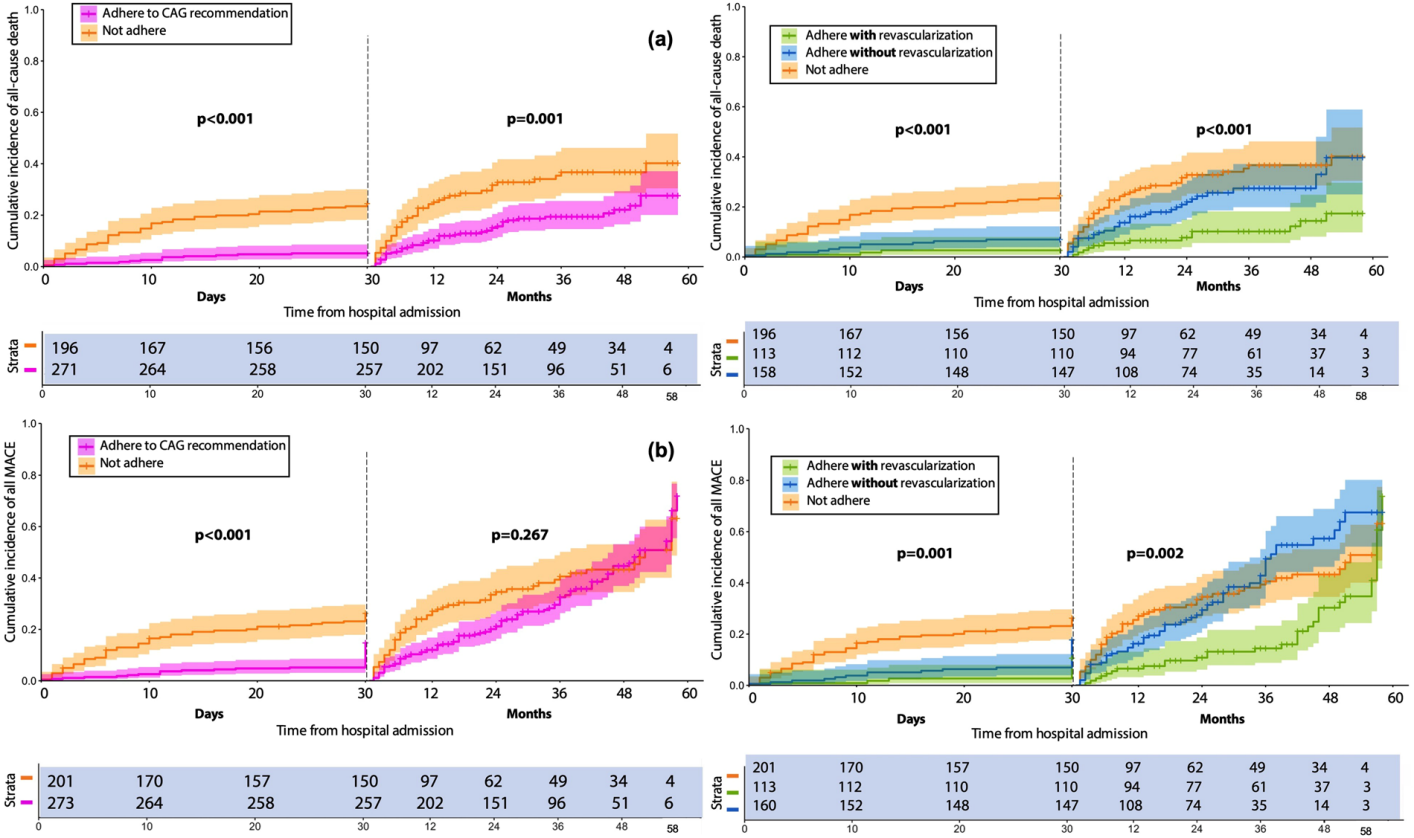
Kaplan-Meier curves describe the short- and medium-term all-cause death (**a**) and composite MACE* (**b**) within: (1) Two groups: Adhere to CAG recommendation vs. Not adhere. (2) Three groups: Adhere with PCI/CABG vs. Adhere without PCI/CABG vs. Not adhere. The difference between groups was estimated using Log-rank test. *First and repeated revascularization (PCI/CABG) as MACE were excluded from the analyses.

The Kaplan-Meier curves better illustrate that the cumulative incidence of all-cause mortality (p = 0.001) and MACE (p ≤ 0.002) were significantly higher in “not adhere” compared to “adhere (with and without revascularization)” patients, both at short- and medium-term follow-up (Fig. 2).

**Figure 3:**
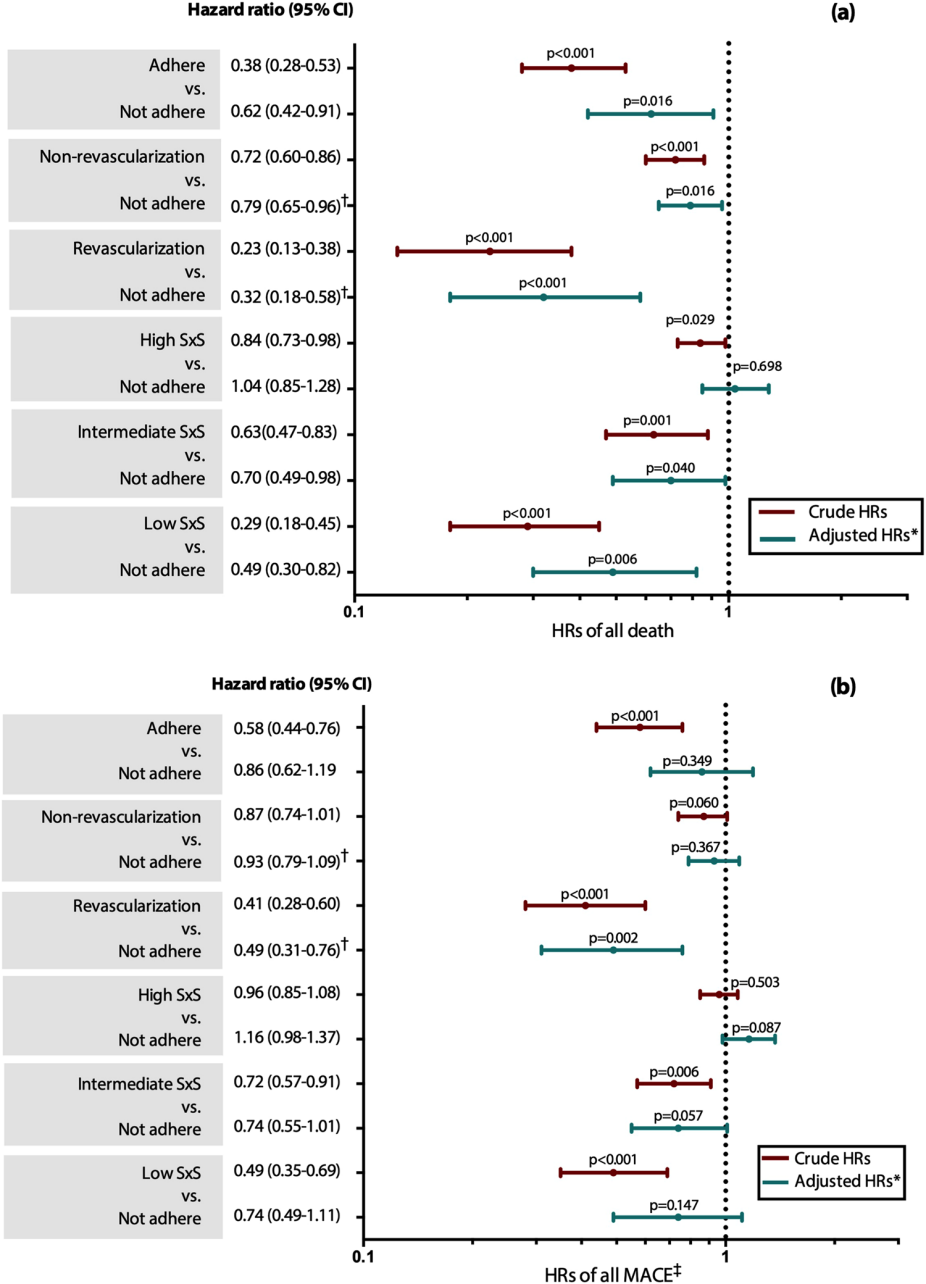
Hazard ratios (HRs) between adhere, revascularization (yes/no), and SYNTAX tertile groups vs. not adhere group for: (**a**) all-cause death and (**b**) composite MACE. *All hazard ratios were adjusted for: age, gender, socio-economic status, education, hypertension, hyperglycemia on admission, eGFR < 60 mL/min, LVEF < 35%, undergoing revascularization (PCI/CABG), adherence on medications, and living on rural area (≥20 kms). ^†^Undergoing revascularization (PCI/CABG) was excluded from these multivariable analyses. ^‡^First and repeated revascularization (PCI/CABG) as adverse outcomes were excluded from the analyses.

Figure 3 describes the forest plot of the crude and adjusted hazard ratios (HRs) comparing the adhere, revascularization, and SYNTAX tertile groups with the “not adhere” counterpart. In crude analyses, adherence to CAG recommendation was associated with a significantly lower all-cause mortality (HR 0.38 (95%CI 0.28–0.53), p < 0.001); and the association remains statistically significant even after adjustment for revascularization (PCI or CABG) and other potential confounders (HR 0.62 (95%CI 0.42–0.91), p = 0.016). In sub-group analysis, adhered patients with revascularization has considerably lower risk of mortality and composite MACE, compared with the not-adhered patients, both in uni- and multivariable analyses; while patients with high SS have comparable outcomes of with the not-adhered patients.

The scheme of insurance or funding of the study population is presented in Table S3. We found that patients with the national health insurance for civil servant (middle to high income) were prone to adhere for undergoing CAG (p < 0.001), while those with lower income holding the national or local insurance tended to refuse this invasive procedure (p < 0.001).

## Discussion

This study showed a low level of compliance (57.6%) with recommendations from guidelines on performing CAG in real-world practice in an underprivileged South-East Asian setting. Patients who did not undergo the procedure were more often older female and had a lower educational and socio-economic level. Adherence to CAG guidelines and subsequent intervention was associated with lower short- and medium-term mortality and morbidity, independent of revascularization status.

After adjustment for potential confounders, the hazard ratio for all-cause mortality attenuated, but remained statistically significant (HR 0.62 (95% CI 0.42–0.91, p = 0.016). Severity of disease and delayed invasive treatment, therefore, remained the dominant factors. Early CAG provides earlier risk stratification, timely revascularization, and accelerates hospital discharge, but greater logistic demands on healthcare system are involved^3^.

A third of patients in our cohort who refused CAG were fearful of undergoing this invasive procedure and were overly concerned about the complications. As an invasive intervention, there are inherent risks and complications related to the procedure that depend on patients’ condition and operators’ skill. Particularly in Indonesian population, patients need to be better informed that major complications associated with this procedure are rare^1^.

Due to poor resources and lack of awareness of symptomatic CAD, we observed a considerable excess delay from symptom onset to admission in this cohort (overall 25.5 (10–48) hours); and consquently, longer delay to an early invasive intervention. While the guidelines refer to an acceptable delay of 2–3 hours in STEMI^12^ and 12–24 hours in NSTE-ACS^3^ as the reference standard. The seriousness of the underlying disease and the extensive comorbidities were influential factors that defer the cardiologist to perform an immediate CAG. Interestingly, previous studies also reported the benefit of deferred CAG^3^. The underlined rationale of delayed or elective angiography is that revascularization may be safer when plaque has been previously stabilized with optimal antithrombotic and/or anti-ischemic agents^3^.

In our unique cohort, because of administrative regulation of the insurance, the low-income patients required to be re-admitted to receive a planned CAG. The inefficiency of the current clinical practice might partly explain the non-adherence to the guidelines recommendation and therefore contribute to the high rate of early deaths in not-adhere group.

Apparently, we also found that non-adhered patients experienced significantly more death within 30 days of admission compared to adhered group (23.9% vs. 5.1%, p < 0.001). The plausible explanations for this finding are: (1) the low rate of early invasive strategy for ACS patients is associated with the increased risk of early mortality; (2) in the absence of a CAG procedure, the cardiologists have less reliable clue to choose an optimal treatment for the patients. The fact that these patients died in the first 14 days of admission (n = 50, 32.3%) was reflecting the more severe and unstable conditions of their coronary disease.

Naturally, a diagnosis found with CAG has to be followed by sensible treatment. Lucas *et al*. have underlined a linear and strong association between the rates of CAG and coronary revascularization, particularly for PCI. For CABG, the association was proved to be modest^13^. Therefore, in the absence of on-site cardiac surgical facilities, a CAG and PCI program for CAD can still have a major impact.

In addition to our main questions to understand the effects (and potential barriers) of undergoing CAG on patients’clinical outcomes, we also investigated the SYNTAX score in patients who underwent a CAG. The SYNTAX score is valuable for decision making and estimating prognosis depending on the treatment instigated: PCI^6,14–17^, CABG^18^, or optimal medical therapy (OMT)^19^. In our study, we found that patients who showed a high SS had comparable outcomes with the not-adhere group.

### Strengths and limitations

The present study is among the first to measure the adherence to the guidelines recommendation to perform CAG in a South-East Asian setting with limited resources, and subsequently reported the impact of that adherence on patients’ short- and mid-term outcomes. In this study, we also aimed to provide evidence that the guidelines for conducting CAG may contribute to equal access and optimal cost-effective treatment and may provide a framework for allocating resources, as well as investing in education of patients and healthcare professionals. Poor registry in most of hospitals in Indonesia required us to actively contact and visit patients’ houses to follow-up the clinical outcomes.

This study has some limitations. The sample size is relatively small. Despite this inherent limitation, we still detected a strong connection between adherence to CAG guidelines and patients’ outcomes both in uni- and multi-variable analyses. Second, we do realize that the SYNTAX score was primarily developed to rate the severity of CAD in view of eligibility for PCI or CABG. The follow-up methods are perhaps unusual by Western standards, but the best that can be achieved in an underprivileged area. However, this study shows the prognostic relevance of undergoing CAG and SYNTAX score, even in populations with poor resources. Last but not least, it could be considered unreasonable to focus on adherence to ACC/AHA or ESC (European Society of Cardiology) guidelines for CAG in Indonesia as these guidelines were developed for application in generously-equipped healthcare systems. Once available, CAG facilities should be more often used to support disease management and decision-making.

## Conclusions

In conclusion, adherence to guidelines on performing CAG in an underprivileged South-East Asian setting is still low. Adherence to these guidelines for undergoing CAG and subsequent intervention is associated with a significantly lower risk of short- and medium-term all-cause mortality. Older age, female gender, low educational and socio-economic levels are associated with the non-adherence. Education for patients and their families, awareness of CVD symptoms, well-established teamwork amongst clinicians, and the infrastructure for invasive intervention should be improved to enhance the adherence to the guidelines standard.

## Supplementary information

Supplementary tables

## Data Availability

All data generated or analysed during this study are included in this published article and its supplementary information files, available from the corresponding author on reasonable request.
