# Supplementary material for: Adherence to guideline recommendations for coronary angiography in a poor South-East Asian setting: Impact on short- and medium-term clinical outcomes
Source: Sci Rep. 2019 Dec 16;9:19163. doi: 10.1038/s41598-019-55299-0 (PMC6915772; doi:10.1038/s41598-019-55299-0)
Supplement: Supplementary file 1 — Supplementary tables [file 41598_2019_55299_MOESM1_ESM.docx]

**Adherence to guideline recommendations for coronary angiography in a poor South-East Asian setting: Impact on short- and medium-term clinical outcomes**

Andriany Qanitha^1,2^*, Cuno SPM Uiterwaal^3^, Jose PS Henriques^4^, Idar Mappangara^5^, Muzakkir Amir^5^, Sumarsono G Saing^6^, Bastianus AJM de Mol^1^

**Supplementary Materials**

| **Classes of recommendation** | |
| --- | --- |
| Class I | Benefit >>> Risk  Procedure/treatment **IS RECOMMENDED** |
| Class IIa | Benefit >> Risk  Additional studies with focused objectives needed  Procedure/treatment **SHOULD BE CONSIDERED** |
| Class IIb | Benefit ≥ Risk  Additional studies with broad objectives needed; additional registry data would be helpful procedure/treatment  Procedure/treatment **MAY BE CONSIDERED** |
| Class III | No benefit/harm  Procedure/treatment **IS NOT RECOMMENDED** |

| **Level of evidence** | |
| --- | --- |
| Level A | Data derived from multiple randomized clinical trials or meta-analysis |
| Level B | Data derived from a single randomized clinical trial or large non-randomized studies |
| Level C | Consensus opinion of experts, case/small studies, retrospective studies, registries, or standard of care |

**Table S1.** Description of classes of recommendation (COR) and level of evidence (LOE) for coronary angiography procedure

| **Table S2.** Guideline recommendations to perform diagnostic coronary angiography (CAG) in our study population | | | | |
| --- | --- | --- | --- | --- |
| **Indications for coronary angiography in CAD** | **Class** | **Frequency (n = 474)** | **Real-world practice** | |
|  |  |  | **Adhere to CAG guidelines**  **(n = 273)** | **Not adhere**  **(n = 201)** |
| **Known or suspected CAD** | | | | |
| - High-risk criteria on non-invasive testing, regardless of severity of angina | IA | 61 (12.9) | 61 (22.3) | 0 (0.0) |
| - CCS class III and IV angina on medical treatment | IB | 16 (3.4) | 14 (5.1) | 2 (1.0) |
| - Patients who have been successfully resuscitated from sudden cardiac death; or have sustained (>30 seconds) monomorphic VT; or non-sustained (<30 seconds) polymorphic VT | IB | 0 (0.0) | 0 (0.0) | 0 (0.0) |
| **Unstable/Non-ST-elevation Acute Coronary Syndrome (NSTE-ACS)** | | | | |
| - An urgent/immediate invasive strategy (diagnostic angiography with intent to perform revascularization) is indicated in UA/NSTEMI patients who have refractory angina or hemodynamic or electrical instability (without serious comorbidities or contraindications to such procedures) | IA | 82 (17.3) | 32 (11.7) | 50 (24.9) |
| - An early invasive strategy is indicated in initially stabilized UA/NSTEMI patients (without serious comorbidities or contraindications to such procedures) who have an increased risk for clinical events | IB | 65 (13.7) | 37 (13.6) | 28 (13.9) |
| - The presence of shock, severe pulmonary congestion, or continuing hypotension | IB | 3 (0.6) | 0 (0.0) | 3 (1.5) |
| **ST-elevation myocardial infarction (STEMI)** | | | | |
| - In candidates for primary or rescue PCI, or as an alternative to thrombolytic therapy in patients who can undergo angioplasty of the infarcted artery within 12 hours of the onset of the symptoms, or >12 hours if ischemic symptoms persist | IA | 164 (34.6) | 94 (34.4) | 70 (34.8) |
| - In patients who are within 36 hours of an acute ST-elevation, Q-wave, or new LBBB MI who developed cardiogenic shock, are <75 years of age, and in whom revascularization can be performed within 18 hours of the onset of the shock | IA | 14 (3.0) | 3 (1.1) | 11 (5.5) |
| - In candidates for surgical repair of ventricular septal rupture or severe MR | IB | 0 (0.0) | 0 (0.0) | (0.0) |
| - In patients with persistent hemodynamic and/or electrical instability | IC | 30 (6.3) | 14 (5.1) | 16 (8.0) |
| **Post-revascularization ischemia** | | | | |
| - Suspected abrupt closure or subacute stent thrombosis after PCI | IB | 3 (0.6) | 3 (1.1) | 0 (0.0) |
| - Recurrent angina or high-risk criteria on non-invasive evaluation within 9 months of PCI | IC | 2 (0.4) | 2 (0.7) | 0 (0.0) |
| **Congestive Heart Failure (CHF)** | | | | |
| - CHF due to systolic dysfunction/acute pulmonary edema with angina or with regional wall motion abnormalities and/or scintigraphic evidence of reversible myocardial ischemia when revascularization is being considered | IB | 34 (7.2) | 13 (4.8) | 21 (10.4) |

| **Table S3.** Insurance or funding scheme of the cohort according to the adherence to CAG recommendation | | | | |
| --- | --- | --- | --- | --- |
| **Insurance or source of funding** | **Adhere**  **(n = 273)** | **Not Adhere**  **(n = 201)** | **Total**  **(n = 474)** | **p-value** |
| **Private**  **Company insurance***  **National insurance for civil servant** (middle to high income – Askes)*  **National or local insurance for low income** (Jamkesmas/Jamkesda)*  **National health insurance (BPJS)**† | 15 (5.5)  8 (2.9)  173 (63.4)  31 (11.4)  46 (16.8) | 21 (10.4)  2 (1.0)  66 (32.8)  83 (41.3)  29 (14.4) | 36 (7.6)  10 (2.1)  239 (50.4)  114 (24.1)  75 (15.8) | 0.050  0.141  <0.001  <0.001  0.434 |
| Values are n (%). Differences were estimated using Pearson’s or exact Chi-square test.  *Before the era of national health insurance.  †Started since January 2014, for all Indonesian citizens.  CAG = coronary angiography; Askes = Asuransi Kesehatan; Jamkesmas = Jaminan Kesehatan Masyarakat; Jamkesda = Jaminan Kesehatan Daerah; BPJS = Badan Penyelenggara Jaminan Sosial. | | | | |
